# Deletion/duplication mutation screening of *TP53* gene in patients with transitional cell carcinoma of urinary bladder using multiplex ligation‐dependent probe amplification

**DOI:** 10.1002/cam4.561

**Published:** 2015-12-21

**Authors:** Mohammad Reza R. Bazrafshani, Pouriaali A. Nowshadi, Sadegh Shirian, Yahya Daneshbod, Fatemeh Nabipour, Maral Mokhtari, Fatemehsadat Hosseini, Somayeh Dehghan, Abolfazl Saeedzadeh, Ziba Mosayebi

**Affiliations:** ^1^ Genetics DepartmentKerman University of Medical SciencesKermanIran; ^2^ Department of PathologyKerman University of Medical SciencesKermanIran; ^3^ Department of PathologySchool of Veterinary PathologyShahrekord UniversityShahrekordIran; ^4^ Shefa Neuroscience Research CenterKhatam‐Al‐Anbia HospitalTehranIran; ^5^ Brain and Spinal Cord Injury Research CenterTehran University of Medical SciencesTehranIran; ^6^ Department of CytopathologyResearch Center of Dr. Daneshbod Path LabShirazIran; ^7^ Department of PathologyShiraz University of Medical SciencesShirazIran; ^8^ Department of GeneticsDr. Mohammad‐Reza Bazrafshani LabKermanIran; ^9^ Department of Medical BiotechnologyFaculty of Medical SciencesTarbiat Modares UniversityTehranIran; ^10^ Department of PediatricsChildren's Medical Center HospitalTehran University of Medical SciencesTehranIran

**Keywords:** Deletion, duplication, multiplex ligation‐dependent probe amplification, TP53 gene, transitional cell carcinoma

## Abstract

Bladder cancer is a molecular disease driven by the accumulation of genetic, epigenetic, and environmental factors. The aim of this study was to detect the deletions/duplication mutations in *TP53* gene exons using multiplex ligation‐dependent probe amplification (MLPA) method in the patients with transitional cell carcinoma (TCC). The achieved formalin‐fixed paraffin‐embedded tissues from 60 patients with TCC of bladder were screened for exonal deletions or duplications of every 12 *TP53* gene exons using MLPA. The pathological sections were examined by three pathologists and categorized according to the WHO scoring guideline as 18 (30%) grade I, 22 (37%) grade II, 13 (22%) grade III, and 7 (11%) grade IV cases of TCC. None mutation changes of *TP53* gene were detected in 24 (40%) of the patients. Furthermore, mutation changes including, 15 (25%) deletion, 17 (28%) duplication, and 4 (7%) both deletion and duplication cases were observed among 60 samples. From 12 exons of *TP53* gene, exon 1 was more subjected to exonal deletion. Deletion of exon 1 of *TP53* gene has occurred in 11 (35.4%) patients with TCC. In general, most mutations of *TP53*, either deletion or duplication, were found in exon 1, which was statistically significant. In addition, no relation between the TCC tumor grade and any type of mutation were observed in this research. MLPA is a simple and efficient method to analyze genomic deletions and duplications of all 12 exons of *TP53* gene. The finding of this report that most of the mutations of *TP53* occur in exon 1 is in contrast to that of the other reports suggesting that exons 5–8 are the most (frequently) mutated exons of *TP53* gene. The mutations of exon 1 of *TP53* gene may play an important role in the tumorogenesis of TCC.

## Introduction

Bladder cancer is a molecular disease resulted from the multistep accumulation of genetic, epigenetic, and environmental factors 27. The human tumor suppressor gene p53, which maps to chromosome 17p13.1, consists of 11 exons spanning over 20 kb of DNA and encodes a 393 amino acid protein. *TP53* gene is the most common target in human tumors 15. The ability of *TP53* gene to manage apoptosis versus DNA repair is not only important for prevention of malignancy but also could be targeted for therapeutic strategies. Mutations in the *TP53* tumor suppressor gene are found at high frequency in a wide range of human cancers 7. *TP53* gene codes for a protein that acts as a transcription factor and serves as a key regulator of the cell cycle. Inactivation of p53 by mutations disrupts the cell cycle and may lead to tumor formation 26. Tumors with intact *TP53* respond more efficiently to chemo and/or radiotherapy 1, 2. Bladder cancer is one of the most common cancers worldwide. It is the fourth most prevalent cancer in men and the 11th most prevalent cancer in women in the United States 8. More than 90% of bladder cancers are carcinomas. Some genomic alterations are strongly associated with specific histopathologic tumor features like grade or stage 6. Very few studies have the molecular genetic abnormalities of bladder carcinoma 9, 16. According to 6 mutations or deletions in tumor suppressor genes *TP53* (70%), *RB* (37%), and *PTEN* (35%) are commonly detected in urothelial carcinoma in situ which are frequently accompanied by chromosomal deletion chromosome 6. Similar studies on molecular genetics pathways of transitional cell carcinoma (TCC) have focused on *TP53* gene mutations as a whole without revealing the role of each exon in carcinogenesis of this kind of neoplasm 4, 16, 17, 21. Mutations of p53 are detected in many cancer types among which urothelial cancer is one of the most prevalent. It is not entirely what is the place and additive value of the current technique as compared to state‐of‐the‐art next‐generation sequencing. More background information on the type of p53 aberrations (deletion, mutation, duplication) occurring in Urothelial cell carcinoma is required to facilitate the interpretation of the data presented.

Multiplex ligation‐dependent probe amplification (MLPA) has recently been developed as a novel method to detect exonic duplication/deletion mutations in multiple human disease genes 12. This method utilizes an oligonucleotide ligation assay with embedded universal primer sequences to permit relatively uniform amplification of multiple (up to 40) regions of the genome revealing the accurate copy number of the regions of interest 12. MLPA technique has been applied in this study for comprehensive analysis of all exons of *TP53* to screen deletion/duplication mutations. In addition, role of each exon in the pathogenesis of TCC and the relation between TCC tumor grade and any type of mutation are reported for the first time in this study.

## Material and Methods

### Samples

Sixty urothelial carcinoma biopsy specimens were obtained from the pathology department of Shafa Hospital, Kerman, Iran. Samples were part of the pathology archive of the Shafa hospital and they have been formalin‐fixed and embedded in paraffin using standard techniques. Grading of the bladder cancer (grade I–IV) was performed by three expert pathologists based on the WHO and ISUP guideline 18, 20.

### DNA extraction

DNA was extracted from formalin‐fixed paraffin‐embedded (FFPE) samples using QIAamp DNA FFPE Tissue Kit (Qiagen, Hilden, Germany) according to the manufacturer's instructions. DNA concentrations were determined using nanodrop.

### Multiplex ligation‐dependent probe amplification

All extracted DNA samples were analyzed for exonic deletions, duplications, and both deletions and duplications affecting *TP53* gene on 17p13.1 using the SALSA P056‐A2 *TP53* MLPA kit (MRC Holland, Amsterdam, the Netherlands), according to the manufacturer's recommendations. The SALSA MLPA kit contains probes for each of the 12 exons of *TP53* gene located on 17p13.1, as well as several probes binding to regions close to telomeric and centromeric of the TP53 gene. Two of the probes detect sequences in exon 1 of *TP53*.

MLPA Products were separated by capillary electrophoresis on the ABI3100 genetic analyzer (Life Technology, Applied Biosystems, Foster City, CA) and analyzed using GeneMarker v. 1.60 software (Softgenetics, LLC, State College, PA).

The peak heights of the samples were compared with control probes and the ratio of peaks were calculated for all exons. If the dosage quotient was 1.0, the results were considered as normal. Thresholds for deletions and duplications were set at 0.5 and 1.5, respectively.

### Statistical analysis

Data analysis was carried out using the Statistical Package for Social Science (SPSS Inc., Chicago, IL, USA) version 16 for windows. Pearson chi‐square and Fisher's exact test were used for comparisons between categorical variables. The level of significance for all tests was set at *P *<* *0.05.

## Results

**Figure 1:**
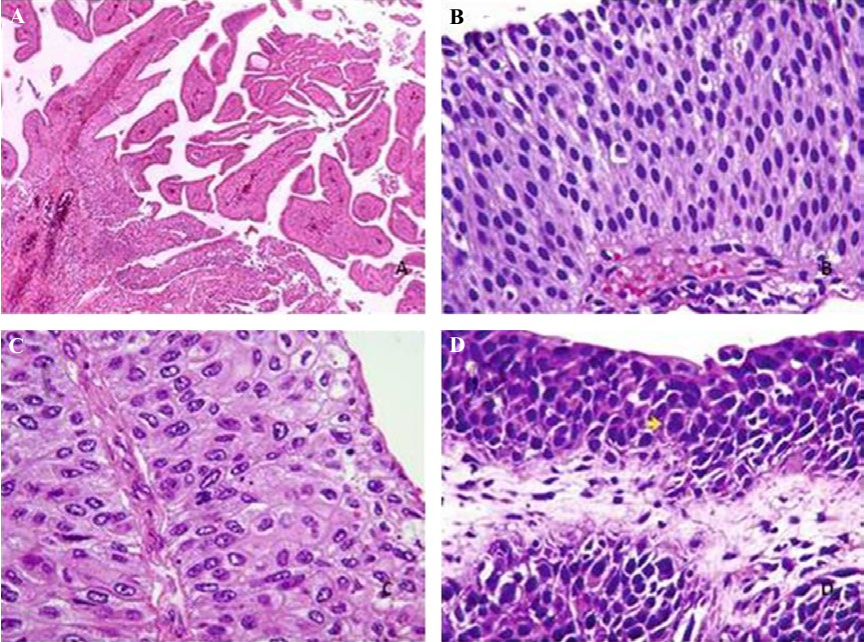
(A) Grade I or papilloma; (B) grade II, papillary neoplasm with low grade of malignancy; (C) grade III, low grade of papillary carcinoma; (D) grade IV, high grade of papillary carcinoma.

The achieved FFPE tissues from 60 patients with TCC of bladder were screened for *TP53* exonal deletions and duplications. The mean age of the patients whose samples were used in this research was 61 years old, 46 (77%) male and 14 (23%) female. The pathological sections were examined by three pathologists according to the WHO scoring guideline and grade I, II, III, and IV of TCC (Fig. 1A–D) were diagnosed in 18 (30%), 22 (37%), 13 (22%), and 7 (11%) cases, respectively.

Based on the grade of the TCC, most of the deletions were observed in grade II samples, followed by grades I, III, and IV (Table 1). While more duplications detected in grades I and II samples rather than grades III and IV (Table 1).

**Table 1:** Exonal mutations in all of 12 exons of TP53 gene in the different grades of TCC

Grade I of TCC (18 patients, 30%, normal patients: 5)												
Mutations/exons	1	2	3	4	5	6	7	8	9	10	11	12
Deletion	4 Ps	–	–	–	1 P	–	–	–	–	–	2 Ps	2 Ps
Duplication	1 P	–	–	–	–	2 Ps	–	3 Ps	–	–	–	–
Grade II of TCC (22 patients, 37%, normal patients: 9)												
Deletion	4 Ps	–	–	2 Ps	1 P	–	–	–	2 Ps	–	1 P	3 Ps
Duplication	2 Ps	–	1 P	–	–	4 Ps	–	1 P	2 Ps	–	–	–
Grade III of TCC (13 patients, 22%, normal patients: 7)												
Deletion	1 P	1 P	–	1 P	1 P	–	–	–	1 P	–	–	–
Duplication	1 P	–	–	–	–	1 P	2 Ps	–	–	–	–	–
Grade IV of TCC (seven patients, 11%, normal patients: 3)												
Deletion	2 Ps	–	1 P	–	–	–	–	–	–	–	1 P	–
Duplication	–	–	–	–	–	–	1 P	2 Ps	1 P	–	–	–
Total deletion	11	1	1	3	3	–	–	–	3	–	4	5
Total duplication	4	–	1	–	–	7	3	6	3	–	–	–

Some of patients had both deletion and duplication or had more than one mutation. TCC, transitional cell carcinoma; P, patient.

**Figure 2:**
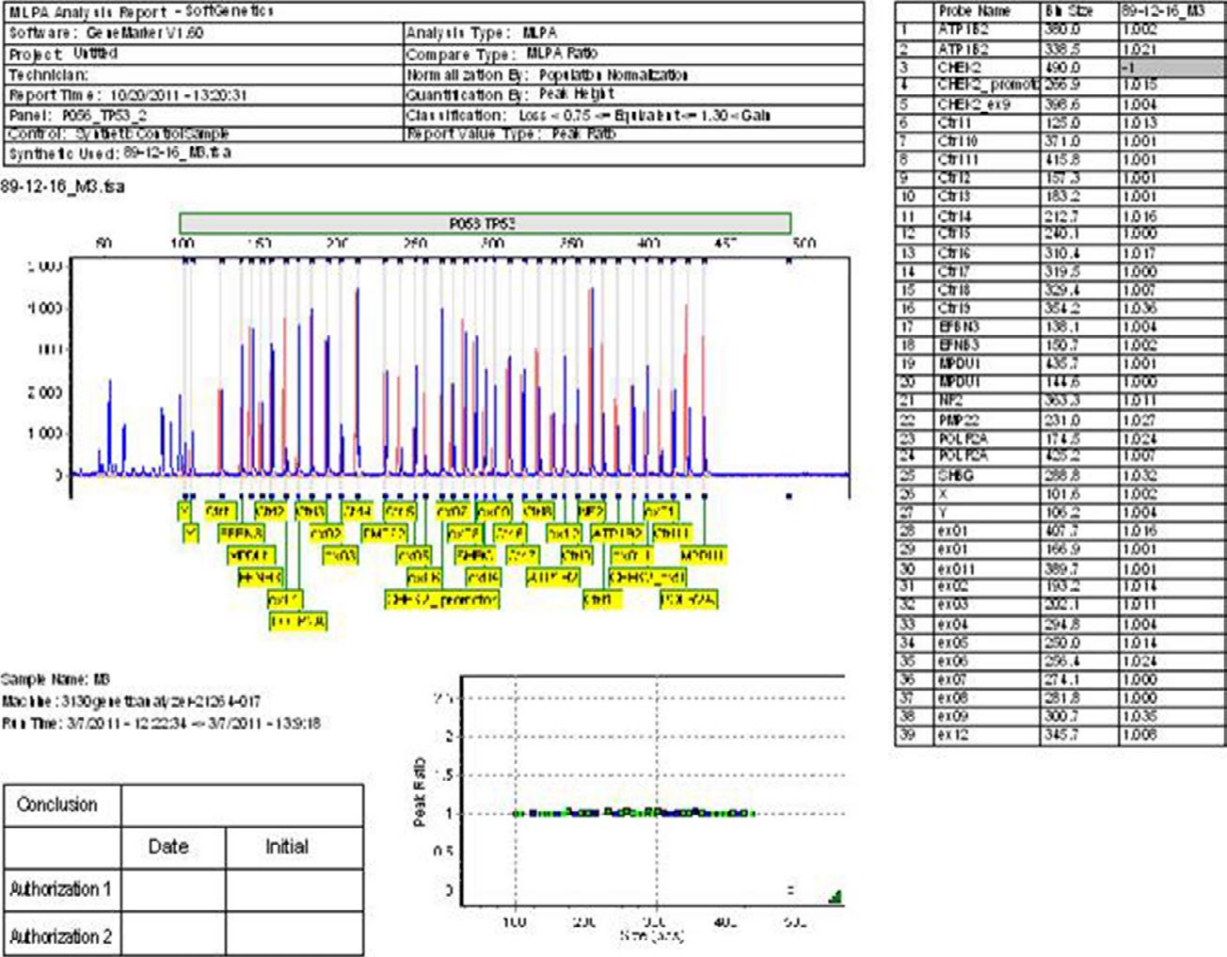
Peak pattern evaluation of *TP53* gene in the normal human using multiplex ligation‐dependent probe amplification (MLPA) test shows the peaks of all exons are in normal range (0.75–1.3).

**Figure 3:**
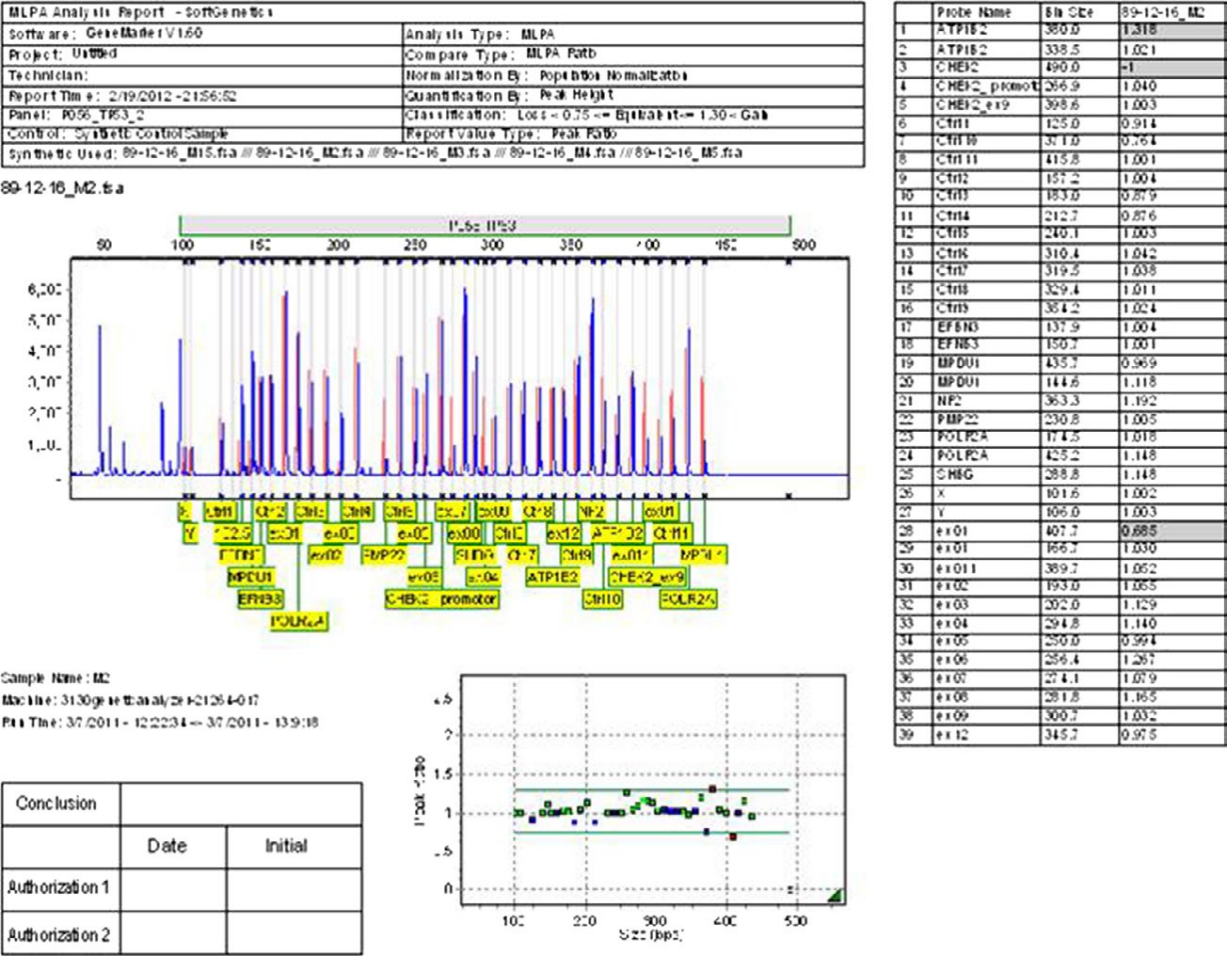
The multiplex ligation‐dependent probe amplification (MLPA) data shows a ratio analysis format where the *X* axis represents fragment size in base pairs, and the *Y* axis represents the probe‐height ratio. Peak pattern evaluation of *TP53* gene in a patient with deletion on exon 1 is represented by a black horizontal line (0.685) which is smaller than that of normal range (0.75–1.3).

**Figure 4:**
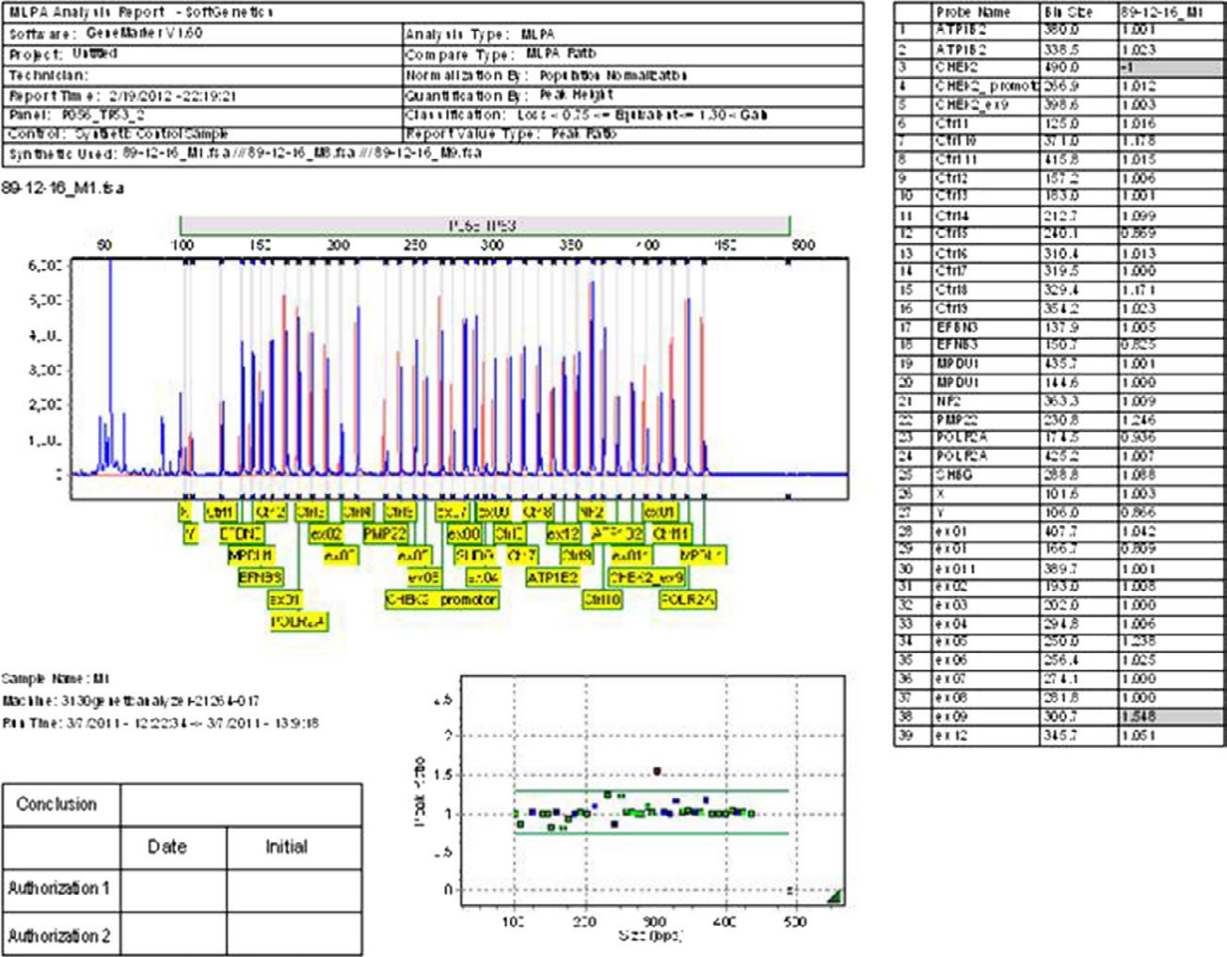
The multiplex ligation‐dependent probe amplification (MLPA) data shows a ratio analysis format where the *X* axis represents fragment size in base pairs, and the *Y* axis represents the probe‐height ratio. Peak pattern evaluation of *TP53* in a patient with duplication on exon 9 is represented by a black horizontal line (1.548) which is larger than that of normal range (0.75–1.3).

None mutation changes of *TP53* gene were detected in 24 (40%) of the patients (Fig. 2). However, other 36 patients (60%) showed mutation changes including, deletion (15 cases, 25%) (Fig. 3), duplication (17 cases, 28%) (Fig. 4) and both exonal deletion and duplication (four cases, 7%). Among 15 cases with exonal deletions; highest number of deletions were four simultaneous deletions in one case followed by three deletions in two cases; two deletions in three cases and one deletion in nine cases. Whereas, among 17 cases with exonal duplication; duplication of one nucleotide was detected in 15 patients and the remaining two patients had two co‐duplications.

Two of the patients with both exonal deletion and duplication carried two deletions and a duplication, one had a deletion as well as two duplications and the other patient had one deletion and a duplication. All mutations showed heterozygote pattern.

Most cases of duplication were detected in exons 6 (7); 8 (6), 1 (4), 7 (3), 9 (3), and 3 (1), while there were no duplication in exons 2, 4, 5, 10, 11, and 12 of *TP53* gene (Table 1). Exonal deletions were detected in exon 1 (11 cases), 12 (5 cases) 11 (4 cases), each of exons 4, 5, and 9 in three cases and each of exons 2 and 3 in one case (Table 1). The highest rate of exonal deletion in *TP53* gene in patients with TCC was detected in exon 1, observed in 11 patients (35.4%). which was statistically significant compared to control group (*P *=* *0.019). Furthermore, most of the mutations either deletion or duplication were found in exon 1, which was statistically significant (*P *=* *0.001) compared to control group, followed by exons 6, 8, 9, 12, 11, 7, 5, 4, 3, and 2.

## Discussion

The results of this research showed that 60% of the examined patients with TCC carried deletion/duplication mutation changes in at least one or more of the 12 exons of *TP53* gene. Most of the patients carried duplication of *TP53* gene followed by deletion and both exonal deletion and duplication. We have found that most cases of the duplication and deletion occurred in exons 6 and 1 of *TP53* gene. However, either of deletion or duplication was detected in all 12 exons of *TP53*, except exon 10. In most of the patients carrying exonal duplication, duplication of only one nucleotide was detected. However, two co‐duplications were observed in two of the patients with exonal duplication. The *p53* gene, located on chromosome 17p13.1, encodes a nuclear phosphoprotein. Originally, *p53* was thought to be a dominant oncogene as it was found to transform rodent fibroblasts in cooperation with the Ras oncogenes. However, further investigations have revealed that *p53* is a tumor suppressor gene. Generally, in the case of *p53*, one allele is lost through a chromosomal deletion and the second allele undergoes some types of intragenic mutations. Chromosomal losses at 17p13 have been reported in a variety of human tumors, including colon, breast, lung, and mesothelioma. Sidransky et al. 25 observed that 61% of invasive cases of bladder cancer carried *p53* mutations, suggesting a role for *p53* in bladder cancer progression 25. TCC is a type of cancer, typically occurs in the urinary system, kidney, urinary bladder, and accessory organs. TCCs are often multifocal, with 30–40% of patients having more than one tumor diagnosed 22. The pattern of growth in TCCs can be papillary, sessile (flat) or carcinoma‐in situ. Despite being superseded by the 2004 WHO grading system (papillary neoplasm of low malignant potential, low grade, and high grade papillary carcinoma), the most commonly used guideline for grading TCCs is the 1973 WHO grading system (papilloma, G1, G2, or G3) used 24. TCC is typically a tumor of older patients, with the average age of presentation being 65 years old. There is a strong male predilection (Male:Female = 4:1) (Leder et al. 1990) 13. In this study, the average age of patients with TCC were 61 years old with a male to female ratio of 46 to 14. We have screened 60 patients with TCC for mutations of all 12 exons of *TP53* using MLPA. Several methods have been used for detection of *p53* mutations. Immunohistochemistry is the simplest approach to detect mutations of *p53* 5. Over 70% of mutations in *p53* exons generate stop codons or frame shifts; therefore, they are more likely to be missed using immunohistochemistry screening 19. Early studies on coding region of *p53* gene (exons 2–11), noted that most mutations occurred in exons 5 and 8 3, 19, 23. In 2001, Berggren et al., 3 screened 189 patients with urinary bladder neoplasms for mutations in exons 5–8 of *p53* gene. They have reported 82 (44%) neoplasms were lowly malignant and 106 (56%) were highly malignant. In a study carried out by 28 on 28 cases of bladder TCC at grade II and III (WHO scale), 13 (46%) patients carried mutations of *TP53* gene. They have shown that codons 280 and 285 of *TP53* may act as hot spots in pathogenesis of bladder TCC 28. By using MLPA method, we could simply screen all exons of *p53* gene, including exon 1, for deletions and duplications in TCC samples. Our results showed that the most frequent *p53* mutations in TCC patients were those detected in exon 1. Several studies have supported this hypothesis that the sequence of human *p53* exon 1 may play an important role in the regulation of *p53* gene expression at the translational level 10, 11. Liu and Bodmer (2006) analyzed all *TP53* exons on a panel of 56 colorectal cancer cell lines and found a 113‐bp homozygous deletion of exon 1 in cell line SW1222. They concluded that exon 1 of *TP53* has been overlooked in nearly all the studies examined the mutation detection. Furthermore, they have emphasized on the need of sequencing every exon of a candidate gene in order to assure detecting all the present mutations in a tumor 14. We demonstrated that MLPA is a sensitive technique to detect genomic deletion/duplication mutations in TCC. Esrig et al. 5 postulated that the amount of *TP53* protein accumulation within the nucleus directly correlates to gene mutation, tumor stage, and tumor grade. Although, most duplications have been reported to be detected in grades I and II TCC tumors as well as deletions in grade II, we found no significant correlation between tumor grades and any types of mutations in this study. This could be due to the small size of the samples and low number of participants. However, the MLPA approach used in this research increased the accuracy of the study.

## Conclusion

In conclusion, MLPA is a simple and efficient method to analysis genomic deletions and duplications in all 12 *TP53* exons. Unlike other reports to date that have supported the exons 5–8 as the most mutated exons of *TP53*, we found that most of the mutations in *TP53* occurred in exon 1in patients with TCC. These mutations may play an important role in the tumorogenesis of TCC. In addition, no significant correlation between different types of mutations in *TP53* and tumor grades was found in our study.

## Conflict of Interest

None declared.
